# The Contributions of Trace Elements on Molecular Subtype-Specific Colorectal Cancer

**DOI:** 10.7150/jca.81686

**Published:** 2023-05-21

**Authors:** Dong-Xiao Bai, Jian-an Xiao, Tian-Chen Huang, Zhi-Ling Shen, Lei Li, Fei-Fei Ding, Ming Wen, Shou-Xin Wu, Xiao-Chen Liu, Hui-Hui Jiang

**Affiliations:** 1 The Fourth Department of General Surgery, Anyang Tumor Hospital, The Affiliated Anyang Tumor Hospital of Henan University of Science and Technology, Anyang 455000, China.; 2 Zhangjiang Center for Translational Medicine, Shanghai Biotecan Pharmaceuticals Co., Ltd., Shanghai 200021, China.; 3 Department of General Surgery, Affiliated Three Two Zero One Hospital of Xi'an Jiaotong University, Hanzhong 723099, China.

**Keywords:** colorectal cancer, blood, KRAS mutations, microsatellite instability, trace elements

## Abstract

**Purpose:** Although growing studies have reported the disturbances of trace elements (TEs) homeostasis was closely associated with the occurrence of colorectal cancer (CRC), the clinical value of TEs in CRC with different molecular subtypes was largely unknown. This study aimed to explore the correlation between *KRAS* mutations/MSI status and serum TEs levels in patients with CRC.

**Methods:** The serum concentrations of 18 TEs were detected by inductively coupled plasma emission spectrometry (ICP-MS). MSI status (two mononucleotides: BAT25, BAT26, three dinucleotides: D2S123, D5S346, and D17S250), *KRAS* (G516T, G517A, G518C, G520T, G521A, G522C, and G532A) mutations were detected by the multiplex fluorescent PCR and the real-time fluorescent quantitative PCR, respectively. The correlations among *KRAS* mutations/MSI status, demographic and clinical characteristics, and TEs were analyzed by Spearman correlation analysis.

**Results:** The propensity score matching (PSM) analysis was adopted to minimize differences between groups. Before PSM, 204 CRC patients were recruited in this study, including 123 *KRAS*-negative patients and 81 *KRAS*-positive patients according to the test results of *KRAS* mutations, and 165 MSS patients and 39 MSI patients based on MSI detection. After PSM, the serum concentration of Mn was significantly lower in CRC patients with *KRAS* mutations than those without *KRAS* mutations, and a significant negative correlation was observed between Mn and Pb in the *KRAS*-positive cases. CRC patients carrying MSI had a significantly lower level of Rb compared to MSS patients. Importantly, Rb was significantly positively correlated with Fe, Mn, Se, and Zn in patients with MSI. Collectively, all our data indicated that the occurrence of different molecular events might be accompanied by different alterations in types and levels of serum TEs.

**Conclusions:** CRC patients with different molecular subtypes presented different alterations in types and levels of serum TEs. Mn was significantly negatively correlated with the *KRAS* mutations, and Rb was noticeably negatively correlated with the MSI status, indicating certain TEs might contribute to the pathogenesis of molecular subtype-specific colorectal cancer.

## Introduction

Colorectal cancer (CRC) is the fourth most common cancer and the third leading cause of cancer-related death globally 1. Morbidity and mortality are steadily increasing in developing nations. Currently, three signaling pathways were closely associated with the occurrence and progression of CRC, including chromosome instability (CIN), microsatellite instability (MSI), and cytosine preceding guanine island methylator phenotype. MSI is characterized by the extensive length variations of microsatellite sequences, which results from the somatic and/or germline mutations existing in one of the DNA mismatch repair genes accompanied by a circumstance of genetic hyper-mutability 2. With growing studies, MSI is not only applied for screening Lynch syndrome (LS) but also regarded as a predictive marker for 5-FU based adjuvant chemotherapy and immunotherapy. However, the application of MSI is highly dependent on the proper validation of MSI status clinically, and irreproducible and inaccurate results will result in an erroneous diagnosis, therapeutic intervention, and prognosis. Although immunohistochemistry (IHC) staining and polymerase chain reaction (PCR)-based tissue tests have shown a high association of MSI, the consistency between ctDNA and tissue was discrepant based on the methodology of tissue test (IHC: 83.0 %, PCR: 97.4 %, NGS: 98.0 %) 3. Consequently, both improving the detected consistency of different test methodologies and exploring auxiliary diagnostic markers to verify MSI status were all needed, especially for colorectal patients without available tumor tissue.

Alongside the established genetic contributors, environmental factors, such as trace elements (TEs), are also regarded as possible culprits in tumorigenesis 4, 5. The concentrations of TEs are very low in the human body, ranging from 0.00001% to 0.01%, but their influences are tremendous on bodily functions due to their component and catalytic roles for many enzymes. The imbalance of TEs may lead to cellular injury, DNA damage, and excessive activation of certain signaling pathways in tumorigenesis and progression. For instance, Fe is one of the most abundant TEs, which is required for protein components and enzyme activities performing a variety of biochemical functions. A close correlation between excess Fe and increased cancer incidence has been reported in epidemiological studies, and tight associations between Fe and carcinoma proliferation, metabolism, or metastasis have been demonstrated in experimental studies 6. As a cofactor, Mg is indispensable for more than 600 enzymes, which were involved in cellular homeostasis and metabolic pathways 7, 8. Both epidemiologic and experimental studies have proved the antitumor effects of Mg on multiple cancer (e.g., colorectal, lung, and bladder cancers) 9-12. Moreover, females with lower serum Mg levels showed a higher risk of CRC, but this phenomenon was not observed among males, suggesting the regulatory mechanism of Mg might have sex-selectivity 13. Zn is a fundamental nutritional element and a key component of antioxidant enzymes 14, 15. Zn plays an important role in antitumor immunity mostly due to its involvement in cell immunity of T-lymphocytes 16. As a natural substance with both nutritional and toxicological properties, Se also participates in the composition and structural integrity of many antioxidant enzymes (e.g., glutathione peroxidase, superoxide dismutase, and thioredoxin reductase) 17, and displays antitumor effects in multiple cancer (e.g., colorectal, lung, prostate, and bladder cancers) 18.

Although the causation between TEs and pathogenesis of CRC is still obscured, growing studies have indicated that the distributions of TEs were significantly different between the carcinoma tissues and the para-carcinoma tissues, as well as between the oncology patients and the healthy controls 5, 19-21. The concentrations of Al, Ca, Cu, Cr, Mg, Mn, Sn, and Si were significantly elevated in colorectal tissues than that in adjacent tissue 19, 22-25. Compared with non-CRC patients, the serum concentration of Zn was significantly lower in CRC patients 26, 27. Several studies also have indicated that serum high levels of Fe were closely correlated with the occurrence of CRC, which might result from the higher metabolism rate of Fe and further aggravate intracellular inflammation in cancer cells 28-30. What's more, the serum concentration of Mg in CRC patients was noticeably lower compared with healthy controls, and returned to normal after treatment, indicating the potential values of serum Mg in clinical monitoring and prognosis 31-33. However, although certain TEs have good abilities to be biochemical markers to predict tumorigenesis and progression, their roles in molecular subtype-specific colorectal cancer were largely unknown.

In the present study, *KRAS* (codon 12/13) mutations, MSI status, and the serum concentrations of 18 TEs were all determined in 204 colorectal patients. This study aimed to explore: (i) which of serum TEs is correlated with the *KRAS* mutations or MSI status in patients with CRC, (ii) what kind of interactions is between the TEs associated with *KRAS* mutations or MSI status and certain demographic and clinical characteristics, and (iii) how do they interact with other TEs in patients with *KRAS*-positive CRC or MSI CRC.

## Materials and Methods

### Patients and samples collection

Two hundred and four colorectal patients from the Department of anus and intestine surgery in the Anyang Tumor Hospital were recruited between April 2020 and July 2022. The TNM staging system of the American Joint Committee on Cancer (AJCC) was preferred and the confirmation of the pathological diagnosis was passed by at least three independent pulmonary pathologists. For all cases, genotype analysis was performed, including *KRAS* Codon 12/13, *BRAF* V600E, and MSI detections. All patients written informed consent, and this study was in accord with the Code of Ethics of the World Medical Association (Declaration of Helsinki) 34.

Inclusion criteria: (1) age > 18, (2) first diagnosis of colon cancer or rectal cancer in situ, (3) without any treatments, (4) all diagnoses meet the standards of the National Comprehensive Cancer Network (NCCN).

Exclusion criteria: (1) patients had been subjected to treatments, including radiotherapy, chemotherapy, or targeted therapy; (2) history of toxic or trace elements exposure; (3) patients had suffered from the surgical procedure within the past six months; (4) patients took antioxidants, vitamins, or nutritional supplements; (5) patients had other diseases, including autoimmune disease, diabetes mellitus, gout, hypoglycemia, ketonemia, liver diseases, primary kidney disease, protein-energy malnutrition, thyroid disease, and vitamin A/D deficiency.

### *KRAS* codon 12/13 and *BRAF* V600E detections

*KRAS* (G516T, G517A, G518C, G520T, G521A, G522C, and G532A) and *BRAF* (T1799A) mutations were detected by real-time fluorescent quantitative PCR (RT-PCR). Briefly, DNA from each tumor tissue was extracted by an FFPE whole genome extraction kit (Qiagen, Hilden, Germany). For *KRAS* mutations, the RT-PCR procedure was as follows: 37°C for 10min, 95°C for 5min, 40 cycles of 95°C for 15s, and 60°C for 60s using Human *KRAS* gene detection kit (Wuhan YZY Medical Science and Technology Co., Ltd, China), and the procedure of *BRAF* mutations was as follows: 37°C for 2min, 95°C for 3min, 45 cycles of 94°C for 15s, and 60°C for 35s using *BRAF* mutant detection kit (Beijing SinoMDgene Technology Co., Ltd, China) by ABI 7500 Real-Time PCR System (ABI, USA).

### MSI detection

A microsatellite-instability (MSI) panel was adopted to detect specific microsatellite repeats based on the multiplex fluorescent PCR, which included two mononucleotides (BAT25, BAT26) and three dinucleotides (D2S123, D5S346, and D17S250). Briefly, an FFPE whole genome extraction kit was used to extract DNA from each tumor tissue and its matched blood sample, and the PCR procedure was as follows: 95°C for 10min, 35 cycles of 95°C for 30s, temperature gradient for 30s, 72°C for 30s, and 72°C for 10min. PCR products were kept at 4°C and analyzed using capillary electrophoresis with ABI 3730XL DNA Analyzer (ABI, USA). MSI is defined as at least one of the five markers, including microsatellite-instability-low (MSI-L) with only one of the five markers and microsatellite-instability-high (MSI-H) with more than one marker. A patient without any of the five markers was regarded as microsatellite stability (MSS).

### Trace elements detection

The serum concentrations of 18 trace elements (TEs) were detected by inductively-coupled plasma mass spectrometry (ICP-MS) (Agilent 7800), including Arsenic (As), Boron (B), Calcium (Ca), Cobalt (Co), Chromium (Cr), Cuprum (Cu), Iron (Fe), Magnesium (Mg), Manganese (Mn), Molybdenum (Mo), Nickel (Ni), Plumbum(Pb), Rubidium (Rb), Selenium (Se), Stannum (Sn), Strontium (Sr), Vanadium (V), and Zinc (Zn). ICP-MS is used for multi-elemental capabilities analysis and the detection procedures are detailed in the manufacturer's instructions 35. In simple terms, more than 2ml of whole blood for each patient was centrifuged at 3000rpm for 10min to get the serum, and the serum was stored at -20°C until use.

### Statistical analysis

Categorical variables were expressed as numbers and compared by chi-square test in SPSS 22.0 (IBM, NY, USA). Continuous variables were shown as median with interquartile range (IQR), and compared by the Unpaired T test or the Two-tailed Mann-Whitney U test in GraphPad Prism 6.0 (La Jolla, CA, USA). The propensity score matching (PSM) analysis with the nearest neighbor principle was performed by MatchIt 4.5.2 in R software (R 4.1.0, R Core Team; https://www.R‑Project.org). Correlated analysis among *KRAS* mutations/MSI status, demographic and clinical characteristics, and 18 TEs were analyzed by Spearman correlation analysis in R software. A *P* value of less than 0.05 was statistically significant.

## Results

### Patient characteristics

A total of 204 colorectal patients were recruited in this study, and their demographic and clinical characteristics were summarized in Table 1. According to the results of MSI detection, the number of colorectal patients with MSS and MSI (including MSI-L and MSI-H) was 165 and 39 (16 and 23), respectively. There were no significant differences between the MSS group (n=165) and the MSI group (n=39) in age, height, weight, gender, drinking history, smoking history, family history of cancer, family history of colorectal cancer, tumor differentiation, distant metastasis, liver metastasis, CEA level, CA-199 level, CA-125 level, *KRAS* Codon 12/13 mutations, and *BRAF* V600E mutation, while significant differences in clinical diagnosis (p<0.0001) and anatomical staging (p<0.05) were observed between these two groups (Table 1). To minimize differences between groups, we performed the PSM analysis with the nearest neighbor principle for a 1:1 matching analysis of relevant clinical variables. After PSM, the MSS group consisted of 39 patients, which was consistent with the MSI group (n=39). Expectedly, no significant differences were observed between these two groups in all relevant demographic and clinical variables (Table 2).

### Comparison of TEs and traditional biomarkers between the *KRAS*-positive group and the *KRAS*-negative group

**Figure 1:**
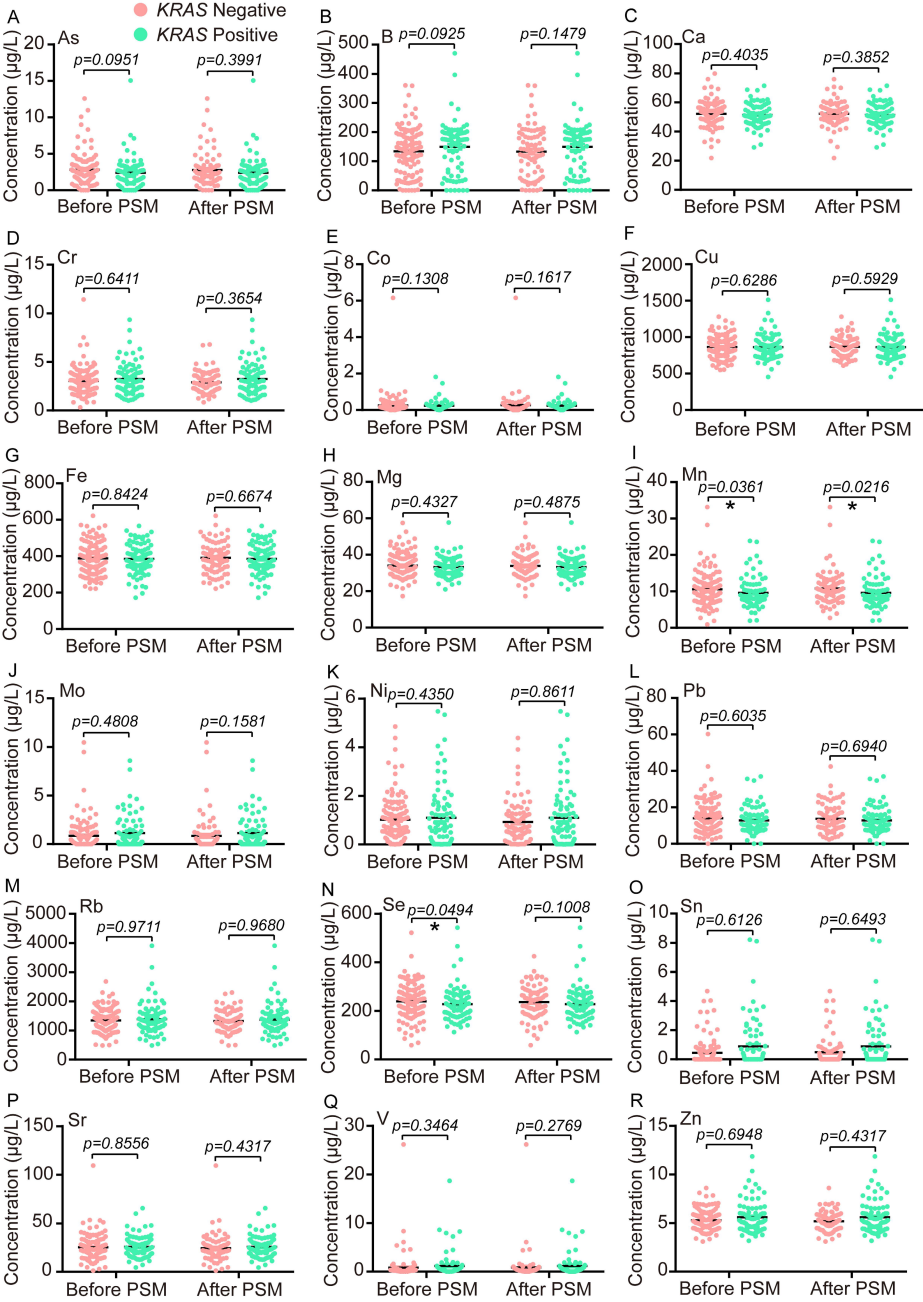
The comparative analysis of 18 trace elements between the *KRAS* positive group and the *KRAS* negative group before or after PSM. Statistical analysis was performed by the Unpaired T test or the Two-tailed Mann-Whitney U test. *p < 0.05.

*KRAS* point mutations are approximately 45% in CRC (https://cancer. sanger.ac.uk/cosmic), and certain positions of codon 12/13 display noticeable carcinogenesis 36. The mutant status of *KRAS* (G516T, G517A, G518C, G520T, G521A, G522C, and G532A) was successfully determined in 204 CRC patients, and the number of *KRAS*-positive cases and *KRAS*-negative cases were 81 and 123, respectively. Since the enrolled patients were below a heterogeneous group, a PSM analysis with the nearest neighbor principle was used to find the different TEs between the *KRAS*-negative group and the *KRAS*-positive group. Before PSM, the serum concentrations of Mn and Se were significantly lower in the *KRAS*-positive group (n=81) than the *KRAS*-negative group (n=123) (Fig. 1I, N), whose median concentration with IQR in the *KRAS*-positive group and the *KRAS*-negative group were as follows: Mn 8.5 (7.165-10.97) μg/L vs. 9.9 (7.64-12.58) μg/L and Se 219.7 (181.4-257.9) μg/L vs. 242.1 (197.1-281.5) μg/L. After PSM, the cohort consisted of 81 *KRAS*-negative samples and 81 *KRAS*-positive samples, and only Mn showed a significant difference between these two groups (Fig. 1I). The median concentration with IQR for Mn after matching in the *KRAS*-positive group and the *KRAS*-negative group was as follows: Mn 8.5 (7.165-10.97) μg/L vs. 10.15 (7.935-12.65) μg/L.

In addition, the serum levels of CEA, CA19-9, and CA12-5 were also examined in 204 of 204, 203 of 204, and 198 of 204 patients, respectively. The normal reference range was 0.00-5.00 ng/mL for CEA, and 0.00-35.00 IU/mL for CA19-9 and CA12-5. For these three traditional biomarkers, no significant differences were observed between the *KRAS*-positive group and the *KRAS*-negative group before or after PSM (Figure S1).

### Comparison of TEs and traditional biomarkers between the MSS group and the MSI group

**Figure 2:**
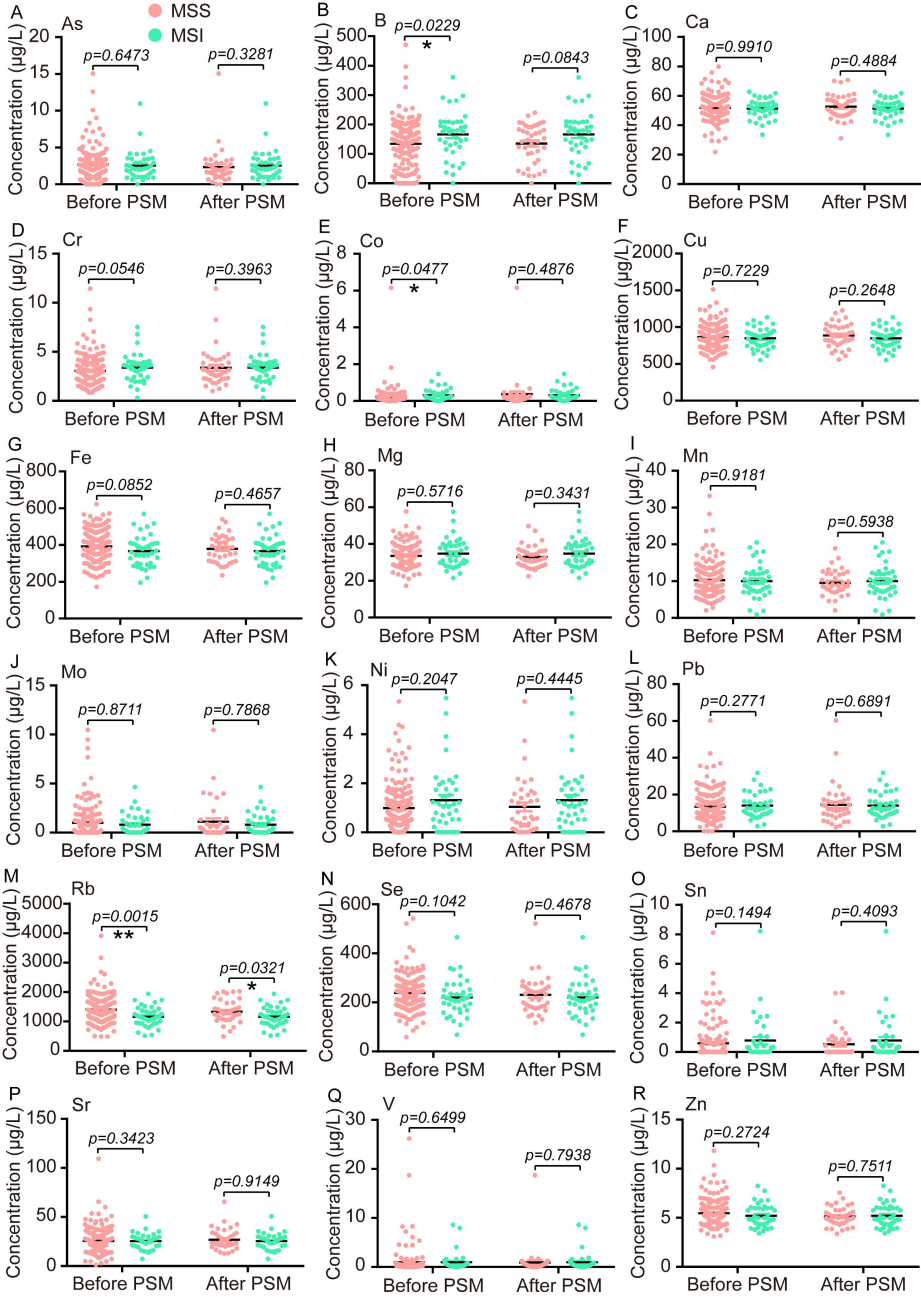
The comparative analysis of 18 trace elements between the MSS group and the MSI group before or after PSM. Statistical analysis was carried out by the Unpaired T test or the Two-tailed Mann-Whitney U test. *p < 0.05, **p < 0.01.

Next, the PSM analysis with the nearest neighbor principle was also performed to find the different TEs between the MSS group and the MSI group. Before PSM, the serum concentrations of B and Co were significantly higher in the MSI group (n=39) than that in the MSS group (n=165), while the serum distribution of Rb presented reverse trends in these two groups (Fig. 2B, E, M). The median concentration with IQR for B, Co, and Rb in the MSS group and the MSI group were as follows: B 145.4 (62.5-193.5) μg/L vs. 175.8 (123.4-209.2) μg/L, Co 0.16 (0.07-0.265) μg/L vs. 0.2 (0.11-0.45) μg/L, and Rb 1332 (1097-1638) μg/L vs. 1143 (936.3-1364) μg/L. After PSM, the cohort consisted of 39 MSI samples and 39 MSS samples, and only the Rb level was significantly lower in the MSI group than the MSS group, 1143 (936.3-1364) μg/L vs. 1278 (1069-1622) μg/L (Fig. 2M). Additionally, we also compared the median concentration with IQR of CEA, CA19-9, and CA12-5 between the MSS group and the MSI group before or after PSM, and no significant differences were found for all three traditional biomarkers (Figure S2).

### Correlations analysis among *KRAS* mutations, demographic and clinical characteristics, and TEs

**Figure 3:**
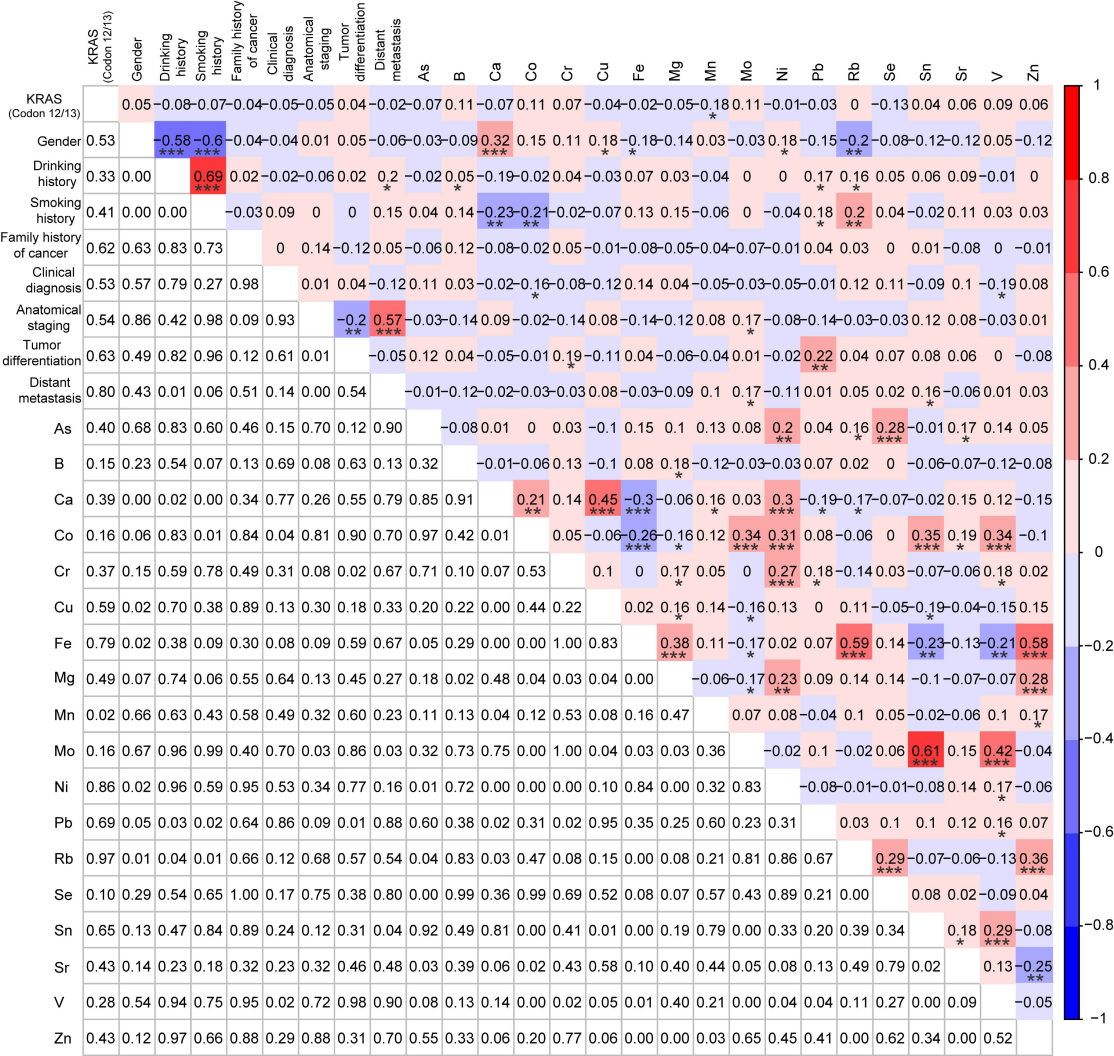
Correlation analysis among *KRAS* mutations, demographic and clinical characteristics, and 18 trace elements for *KRAS*-positive patients (n=81) and *KRAS*-negative patients (n=81) after PSM. *p < 0.05, **p < 0.01, and ***p < 0.001.

Since the information about *KRAS* mutations, demographic and clinical characteristics (gender, drinking history, smoking history, family history of cancer, clinical diagnosis, anatomical staging, tumor differentiation, and distant metastasis), and the serum concentrations of 18 TEs were all acquired from the *KRAS*-positive group (n=81) and the *KRAS*-negative group (n=81), we explored their correlations by Spearman correlation analysis. Mn was significantly negatively correlated with the *KRAS* mutations (r = -0.18, p < 0.05) (Fig. 3). Except for significant correlations between TEs and the demographic characteristics (gender, drinking history, and smoking history), multiple TEs were also significantly correlated with the clinical characteristics (clinical diagnosis, anatomical staging, tumor differentiation, and distant metastasis), such as between tumor differentiation and Pb (r = 0.22, p < 0.01) (Fig. 3). Meanwhile, most of these metals were significantly correlated with others, including between As and Se (r = 0.28, p < 0.001), between Mn and Ca (r = 0.16, p < 0.05), and others (Fig. 3).

**Figure 4:**
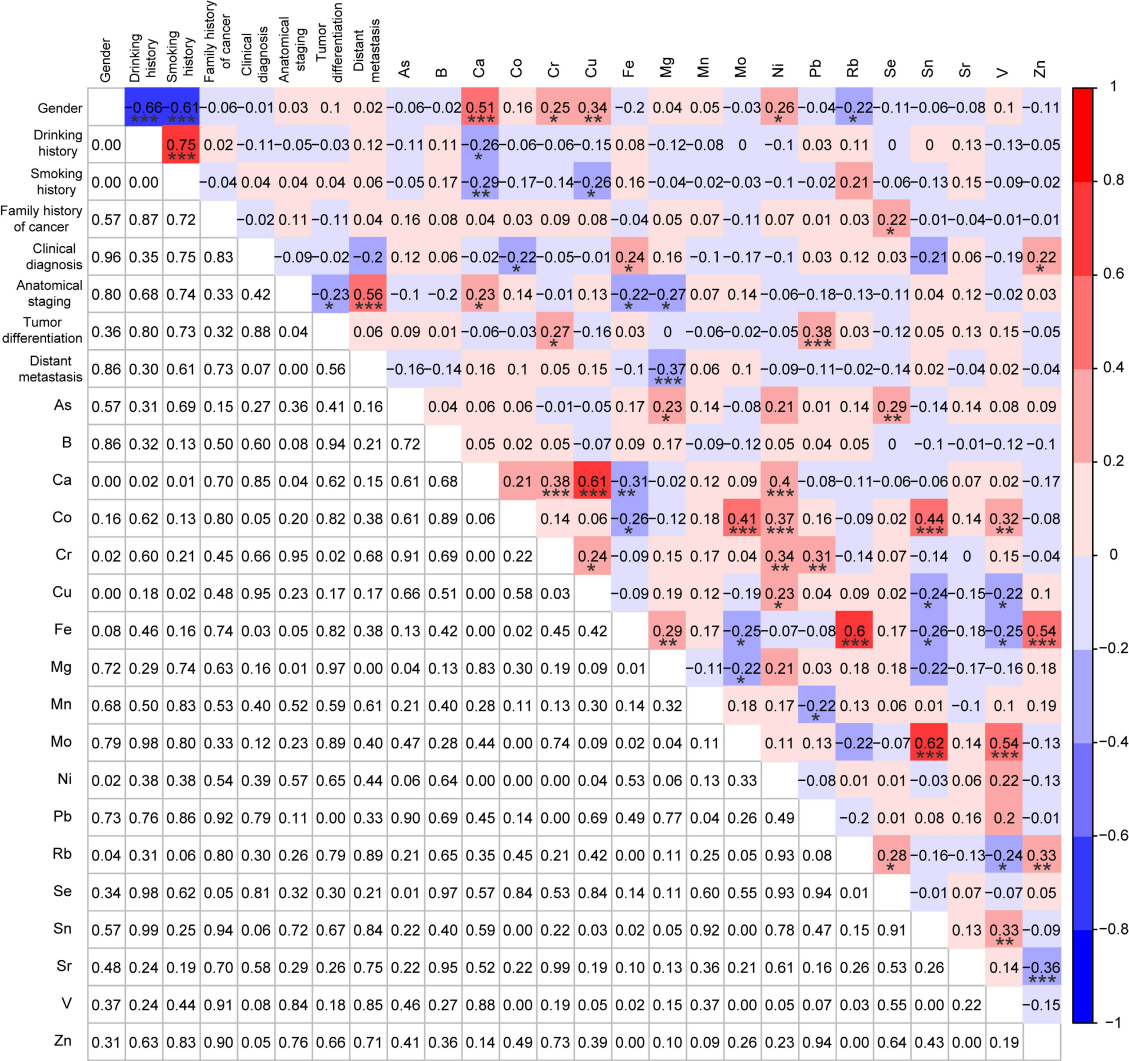
Correlation analysis among demographic characteristics, clinical characteristics, and 18 trace elements in CRC patients with *KRAS*-positive mutations (n=81). *p < 0.05, **p < 0.01, and ***p < 0.001.

To further elucidate the interactive mechanisms of Mn in *KRAS*-positive CRC, the Spearman correlation analysis was used to explore correlations among Mn, demographic and clinical characteristics, and other 17 TEs in the *KRAS*-positive group (n=81). No significant correlations were observed except a significant negative correlation between Mn and Pb (r = -0.22, p < 0.05) (Fig. 4).

### Correlations analysis among MSI status, demographic and clinical characteristics, and TEs

**Figure 5:**
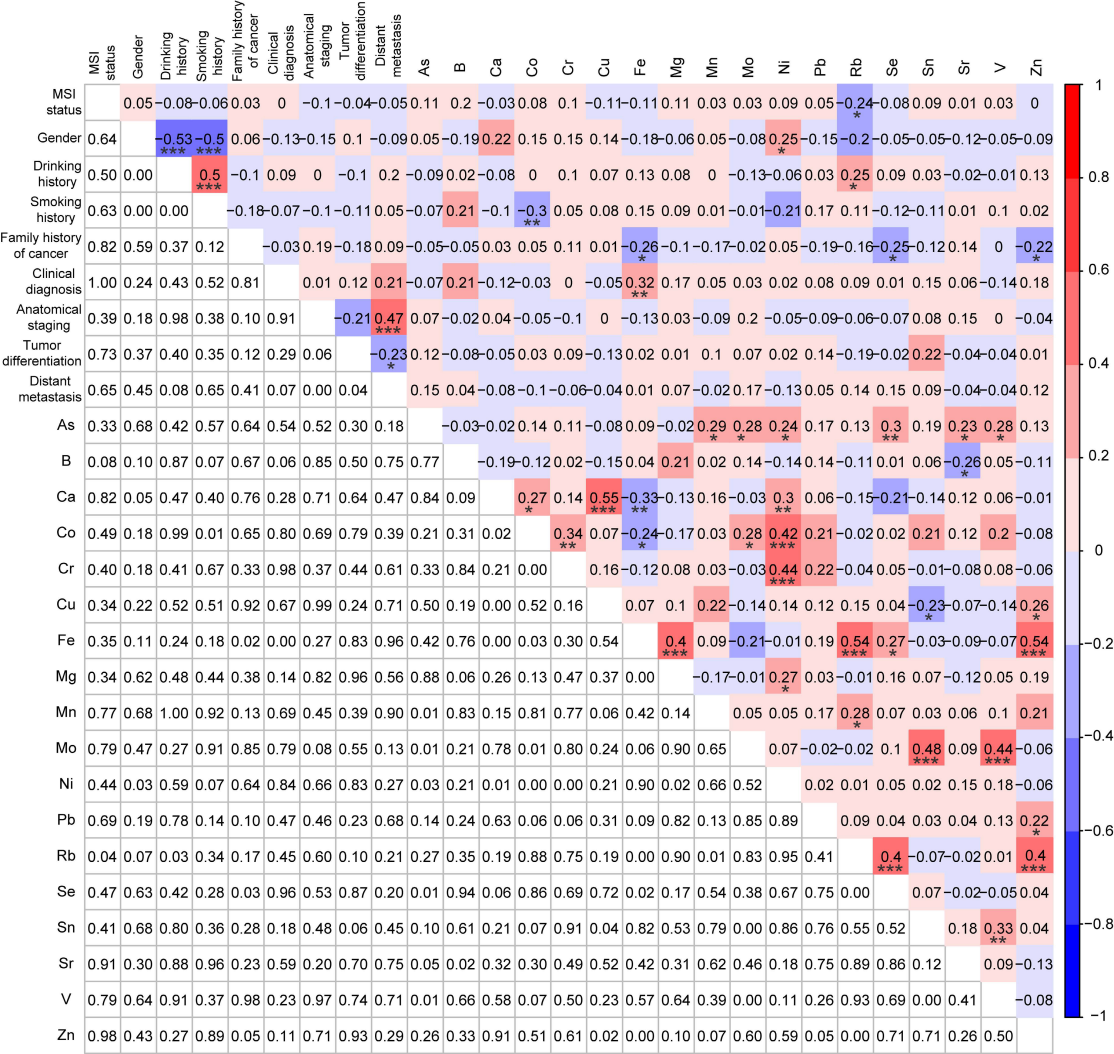
Correlation analysis among MSI status, demographic and clinical characteristics, and 18 trace elements for MSI patients (n=39) and MSS patients (n=39) after PSM. *p < 0.05, **p < 0.01, and ***p < 0.001.

To further explore the correlations among molecular subtypes, demographic and clinical characteristics, and 18 TEs, the Spearman correlation analysis was also performed for the MSI cases (n=39) and the MSS cases (n=39) after PSM. Rb was significantly negatively correlated with the MSI status (r = -0.24, p < 0.05) (Fig. 5). Consistent with the results between patients with and without *KRAS* mutations, the same significant correlations were also observed between the demographic characteristics and TEs (e.g., gender and Ni, drinking history and Rb, smoking history and Co), and in most of these metals (e.g., As and Ni, As and Se, Rb, and Se) (Fig. 5). However, there were no significant correlations between family history of cancer and TEs for patients based on 1:1 *KRAS* mutations matching analysis, while family history of cancer was significantly negatively correlated with Fe (r = -0.26, p < 0.05), Se (r = -0.25, p < 0.05), and Zn (r = -0.22, p < 0.05) for patients with 1:1 MSI matching, indicating the occurrence of different molecular events was associated with different alterations in types and levels of TEs (Fig. 3 and 5).

**Figure 6:**
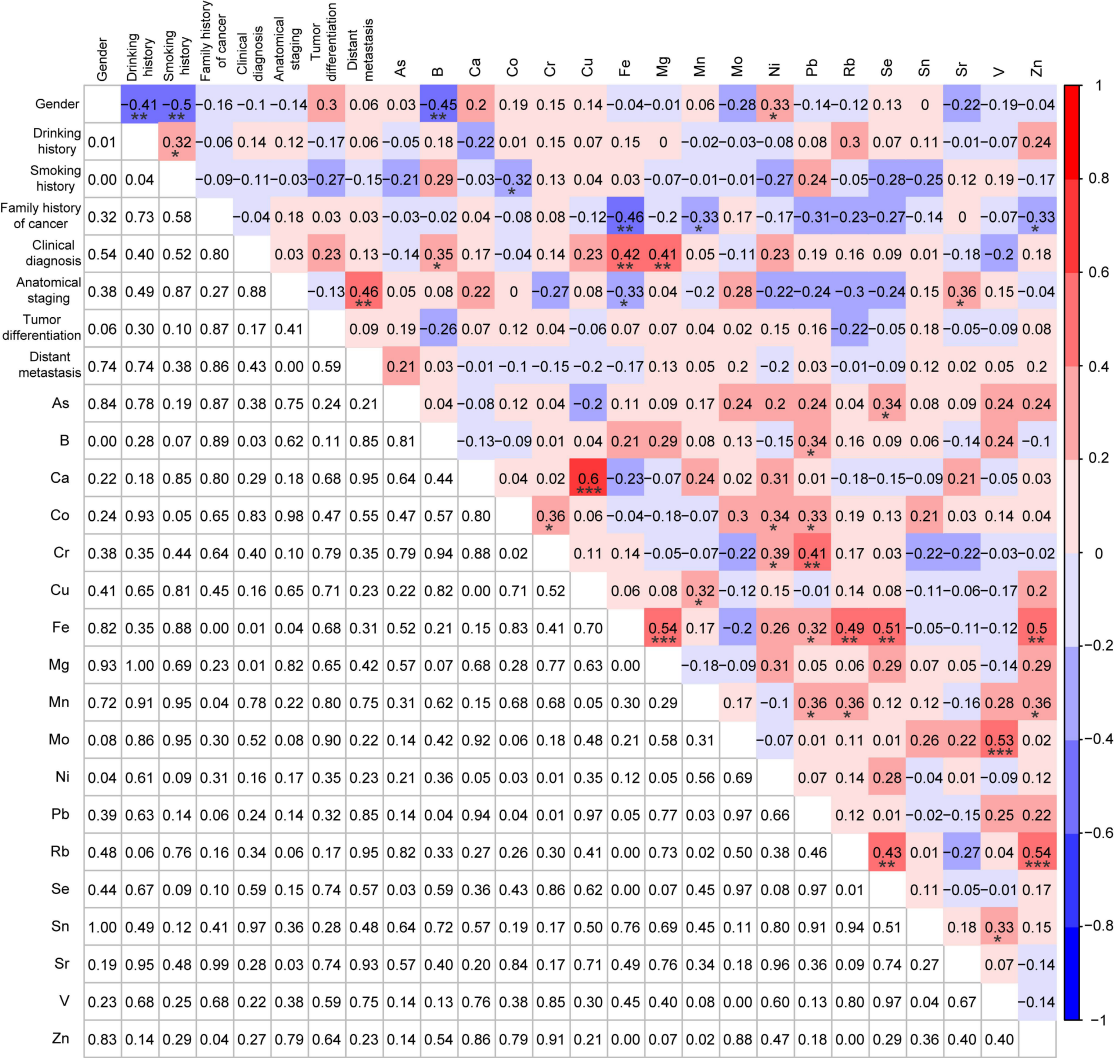
Correlation analysis among demographic characteristics, clinical characteristics, and 18 trace elements in CRC patients with MSI (n=39). *p < 0.05, **p < 0.01, and ***p < 0.001

In addition, the interactions of Rb with other factors in patients with MSI CRC (n=39) were also explored by Spearman correlation analysis. Interestingly, Rb did not show any significant correlations with the demographic and clinical characteristics, while it was significantly positively correlated with Fe (r = 0.49, p < 0.01), Mn (r = 0.36, p < 0.05), Se (r = 0.43, p < 0.01), and Zn (r = 0.54, p < 0.001) (Fig. 6).

## Discussion

Here 204 patients were recruited, including 123 *KRAS*-negative patients and 81 *KRAS*-positive patients according to the *KRAS* mutant test results, and 165 MSS patients and 39 MSI patients based on the detection of MSI status. After PSM, the serum concentration of Mn was significantly lower in CRC patients with *KRAS* mutations than those without *KRAS* mutations, and a significant negative correlation was observed between Mn and Pb in the *KRAS*-positive cases. CRC patients carrying MSI had a significantly lower level of Rb compared to MSS patients. Importantly, Rb was significantly positively correlated with Fe, Mn, Se, and Zn in patients with MSI. Collectively, all our data indicated that the occurrence of different molecular events might be accompanied by different alterations in types and levels of TEs.

Unsupervised clustering of gene alteration profiles has revealed intratumoral heterogeneity, which is useful to identify various molecular subtypes in CRC 37, 38. *KRAS* mutation is the most common variation type accounting for 45%-65% of the total patients with CRC. Although the classic tumorigenesis pathway focusing on *APC*/*KRAS*/*TP53* mutations has been established well in CRC 39, 40, the diverse biochemical properties of *KRAS* mutational isoforms to hydrolyze GTP and activate downstream signaling pathways still challenge scientists and physicians dramatically 41, 42. Moreover, metastatic CRC carrying *KRAS* mutations shows remarkable resistance to cetuximab due to the integrated activation of the RAS-RAF-MEK-ERK pathway 43, 44. Thus, it is interesting to explore the risk factors associated with *KRAS* mutations to elucidate pathogenesis and evaluate prognosis in CRC.

In this study, patients carrying *KRAS* mutations had a significantly lower level of Mn compared to those carrying wild-type *KRAS*. What's more, Mn was significantly negatively correlated with Pb in the *KRAS*-positive cases. To our knowledge, it is the first time to report these findings in Chinese patients with CRC. Recently, *Sajida et al.* has reported that the expression of the Ca^2+^ activated potassium channel KCNN4 (SK4) is significantly enhanced in CRC tissues in comparison with the normal tissues, and KCNN4 expression is much higher in patients with *KRAS*-positive mutations than wild-type patients 45. What's more, SK4 plays a crucial role in accelerating cell migration and invasion via regulating Ca^2+^ entry in HCT116 cells 45. As an essential nutrient, copper is highly needed for cell growth and metastasis in tumor tissues, and copper-related diagnostic methods have been proposed in multiple cancers including CRC 46. The copper (Cu)-exporter ATP7A is upregulated at the *KRAS*-mutated cells' surface and protects these cells from cuproptosis, indicating a *KRAS*-selective vulnerability based on the copper bioavailability in CRC 47. In addition, early hypomagnesemia was a predictor of efficacy and outcome in wild-type *KRAS* advanced colorectal cancer patients receiving cetuximab + irinotecan (CTX+IRI) 48. In mice, compared to selenium from yeast, dairy-selenium noticeably increased plasma selenium levels and acute apoptotic response to azoxymethane (AARGC), and reduced cell proliferation and *Kras* mutation frequency in aberrant crypt foci (ACF), indicating the plasma alterations of certain TEs might reduce the morbidity of cancers with specific molecular subtypes 49. Collectively, although only a few researchers have reported the correlations between *KRAS* mutations and serum TEs accumulations, their specific roles contributing to tumorigenesis, progression, and metastasis in *KRAS* mutant subtypes should not be neglected.

The clinical application of MSI is highly dependent on its accurate detection, and the consistency between ctDNA and tissue or among different test methodologies (e.g., NGS, PCR, and IHC) was still discrepant 50, 51. Thus, besides improving the detection consistency, it is also necessary to complement biochemical alterations closely associated with MSI. In this study, the serum concentration of Rb in patients carrying MSI was significantly lower than that in MSS patients. Importantly, Rb was significantly positively correlated with Fe, Mn, Se, and Zn in patients with MSI. Compared with published papers, these correlations were first reported in Chinese patients. Currently, only a few epidemiologic studies have reported the correlations between the MSI status and TEs for patients with CRC. In a prospective follow-up study for 88,506 women and 47,733 men, calcium intake was strongly associated with CIMP-negative/low and MSS/MSI-L subtypes 52. However, another two case-case studies reported that calcium intake within 5 years before CRC diagnosis did not show significant associations with CIMP subtypes and MSI status 53, 54. The discrepancy might be due to diverse recruited populations including different calcium intake, absorption, and utilization. Meanwhile, a noticeable correlation between the serum concentration of Ca and the MSI status was also not observed in our study. In addition, low cuproptosis (copper-induced cell death) scores were closely associated with high tumor mutation burden, MSI-H, high CTLA4 expression, and high immune cell proportion score, indicating a novel cuproptosis-related molecular pattern to guide individualized treatment 55. Consequently, it is interesting to discover the correlations between TEs and specific molecular subtypes, which not only helps to elucidate the pathogenesis of CRC but also helps to provide novel therapeutic concepts and means.

Still, this study has several limitations. Firstly, healthy controls were not enrolled and only the CRC patients were recruited. The reason for this situation is mainly because our original intention was to explore the biochemical differences of molecular subtype-specific CRC, focusing on the *KRAS*-negative group vs. the *KRAS*-positive group, and the MSS group vs. the MSI group. Consistent with previous studies, more than half of MSI-H patients are diagnosed with colon cancer (86.96%, 20/23) and at Stage II (69.57%, 16/23) 56, indicating the reliability of our data indirectly. Secondly, the detection of 18 TEs was only performed on blood samples without matched tumor tissue for every case in this study, which is mainly because of the limitation of tumor tissue size and amount. On the other hand, the more convenient and less invasive the method, the more popular it will be received for diagnosis and monitoring in clinical. Lastly, the sample size was small, especially for the number of molecular subtype-specific patients, and these results need to be verified by larger sample sizes and multicenter studies.

## Conclusions

CRC patients with different molecular subtypes presented different alterations in types and levels of serum TEs. Mn was significantly negatively correlated with the *KRAS* mutations, and Rb was noticeably negatively correlated with the MSI status, indicating certain TEs might contribute to the pathogenesis of molecular subtype-specific colorectal cancer.

## Supplementary Material

Supplementary figures.

## Figures and Tables

**Table 1:** The characteristics of CRC patients according to MSI status before PSM.

Characteristics	Total (n=204)	MSS (n=165)	MSI (n=39)	*P* value
Gender				
Male	114	90	24	0.429
Female	90	75	15	
Drinking history				
Yes	71	56	15	0.594
No	133	109	24	
Smoking history				
Yes	63	52	11	0.687
No	141	113	28	
Family history of cancer				
Yes	83	66	17	0.682
No	121	99	22	
Family history of colorectal cancer				
Yes	9	5	4	0.123
No	195	160	35	
Clinical diagnosis				
Colon cancer	93	64	29	0.000 ****
Rectal cancer	111	101	10	
Anatomical staging				
Ⅰ	19	16	3	0.018 *
Ⅱ	91	65	26	
Ⅲ	71	63	8	
Ⅳ	23	21	2	
Tumor differentiation				
Low	18	13	5	0.684
Middle-low	19	16	3	
Middle	165	134	31	
High	2	2	0	
Distant metastasis				
Yes	23	21	2	0.286
No	181	144	37	
Liver metastasis				
Yes	17	16	1	0.260
No	187	149	38	
CEA at baseline				
Normal	118	93	25	0.379
Elevated	86	72	14	
CA19-9 at baseline				
Normal	162	131	31	0.887
Elevated	41	33	8	
Unknown	1	1	0	
CA12-5 at baseline				
Normal	189	154	35	0.581
Elevated	9	6	3	
Unknown	6	5	1	
*KRAS* Codon 12/13				
Yes	81	68	13	0.366
No	123	97	26	
*BRAF* V600E				
Yes	3	3	0	1.000
No	201	162	39	
Age (median with IQR)	63 (54-67)	62 (53.5-67)	62.5 (57-68.25)	
Height/m (median with IQR)	1.65 (1.58-1.7)	1.65 (1.58-1.71)	1.6 (1.553-1.678)	
Weight/Kg (median with IQR)	64.51 (56-70.95)	64.6 (56.9-71.5)	60 (56.15-69.55)	

*p < 0.05, ****p < 0.0001.

**Table 2:** The characteristics of CRC patients according to MSI status after PSM.

Characteristics	Total (n=78)	MSS (n=39)	MSI (n=39)	*P* value
Gender				
Male	50	26	24	0.637
Female	28	13	15	
Drinking history				
Yes	33	18	15	0.492
No	45	21	24	
Smoking history				
Yes	24	13	11	0.624
No	54	26	28	
Family history of cancer				
Yes	33	16	17	0.819
No	45	23	22	
Family history of colorectal cancer				
Yes	9	5	4	1.000
No	69	34	35	
Clinical diagnosis				
Colon cancer	58	29	29	1.000
Rectal cancer	20	10	10	
Anatomical staging				
Ⅰ	8	5	3	0.338
Ⅱ	44	18	26	
Ⅲ	21	13	8	
Ⅳ	5	3	2	
Tumor differentiation				
Low	9	4	5	0.729
Middle-low	8	5	3	
Middle	61	30	31	
High	0	0	0	
Distant metastasis				
Yes	5	3	2	1.000
No	73	36	37	
Liver metastasis				
Yes	2	1	1	1.000
No	76	38	38	
CEA at baseline				
Normal	48	23	25	0.642
Elevated	30	16	14	
CA19-9 at baseline				
Normal	64	33	31	0.555
Elevated	14	6	8	
Unknown	0	0	0	
CA12-5 at baseline				
Normal	72	37	35	0.534
Elevated	5	2	3	
Unknown	1	0	1	
*KRAS* Codon 12/13				
Yes	26	13	13	1.000
No	52	26	26	
*BRAF* V600E				
Yes	1	1	0	1.000
No	77	38	39	
Age (median with IQR)	63 (58.75-69.25)	63 (60-70)	63 (56-69)	
Height/m (median with IQR)	1.65 (1.575-1.7)	1.66 (1.58-1.7)	1.65 (1.56-1.7)	
Weight/Kg (median with IQR)	64.5 (55.38-70)	65 (56.8-70)	60 (55-70)	

## References

[1] Sung H, Ferlay J, Siegel RL, Laversanne M, Soerjomataram I, Jemal A (2021). Global Cancer Statistics 2020: GLOBOCAN Estimates of Incidence and Mortality Worldwide for 36 Cancers in 185 Countries. CA Cancer J Clin.

[2] Luchini C, Bibeau F, Ligtenberg MJL, Singh N, Nottegar A, Bosse T (2019). ESMO recommendations on microsatellite instability testing for immunotherapy in cancer, and its relationship with PD-1/PD-L1 expression and tumour mutational burden: a systematic review-based approach. Ann Oncol.

[3] Willis J, Lefterova MI, Artyomenko A, Kasi PM, Nakamura Y, Mody K (2019). Validation of Microsatellite Instability Detection Using a Comprehensive Plasma-Based Genotyping Panel. Clin Cancer Res.

[4] Phipps O, Brookes MJ, Al-Hassi HO (2021). Iron deficiency, immunology, and colorectal cancer. Nutr Rev.

[5] Navarro Silvera SA, Rohan TE (2007). Trace elements and cancer risk: a review of the epidemiologic evidence. Cancer Causes Control.

[6] Torti SV, Manz DH, Paul BT, Blanchette-Farra N, Torti FM (2018). Iron and Cancer. Annu Rev Nutr.

[7] Blaszczyk U, Duda-Chodak A (2013). Magnesium: its role in nutrition and carcinogenesis. Rocz Panstw Zakl Hig.

[8] Kanellopoulou C, George AB, Masutani E, Cannons JL, Ravell JC, Yamamoto TN (2019). Mg(2+) regulation of kinase signaling and immune function. J Exp Med.

[9] Chen Y, Xiao M, Zhao H, Yang B (2015). On the antitumor properties of biomedical magnesium metal. J Mater Chem B.

[10] Yuan Z, Guo G, Sun G, Li Q, Wang L, Qiao B (2022). Magnesium isoglycyrrhizinate suppresses bladder cancer progression by modulating the miR-26b/Nox4 axis. Bioengineered.

[11] Li T, Yu Y, Shi H, Cao Y, Liu X, Hao Z (2020). Magnesium in Combinatorial With Valproic Acid Suppressed the Proliferation and Migration of Human Bladder Cancer Cells. Front Oncol.

[12] Castiglioni S, Maier JA (2011). Magnesium and cancer: a dangerous liason. Magnes Res.

[13] Polter EJ, Onyeaghala G, Lutsey PL, Folsom AR, Joshu CE, Platz EA (2019). Prospective Association of Serum and Dietary Magnesium with Colorectal Cancer Incidence. Cancer Epidemiol Biomarkers Prev.

[14] Yang YW, Dai CM, Chen XH, Feng JF (2021). The Relationship between Serum Trace Elements and Oxidative Stress of Patients with Different Types of Cancer. Oxid Med Cell Longev.

[15] Choi S, Liu X, Pan Z (2018). Zinc deficiency and cellular oxidative stress: prognostic implications in cardiovascular diseases. Acta Pharmacol Sin.

[16] Jouybari L, Kiani F, Akbari A, Sanagoo A, Sayehmiri F, Aaseth J (2019). A meta-analysis of zinc levels in breast cancer. J Trace Elem Med Biol.

[17] Calderon Guzman D, Juarez Olguin H, Osnaya Brizuela N, Hernandez Garcia E, Lindoro Silva M (2019). The Use of Trace and Essential Elements in Common Clinical Disorders: Roles in Assessment of Health and Oxidative Stress Status. Nutr Cancer.

[18] Vinceti M, Filippini T, Del Giovane C, Dennert G, Zwahlen M, Brinkman M (2018). Selenium for preventing cancer. Cochrane Database Syst Rev.

[19] Rinaldi L, Barabino G, Klein JP, Bitounis D, Pourchez J, Forest V (2015). Metals distribution in colorectal biopsies: New insight on the elemental fingerprint of tumour tissue. Dig Liver Dis.

[20] Pavesi T, Moreira JC (2020). Mechanisms and individuality in chromium toxicity in humans. J Appl Toxicol.

[21] Mulware SJ (2013). Trace elements and carcinogenicity: a subject in review. 3 Biotech.

[22] Lavilla I, Costas M, Miguel PS, Millos J, Bendicho C (2009). Elemental fingerprinting of tumorous and adjacent non-tumorous tissues from patients with colorectal cancer using ICP-MS, ICP-OES and chemometric analysis. Biometals.

[23] Gupta SK, Shukla VK, Vaidya MP, Roy SK, Gupta S (1993). Serum and tissue trace elements in colorectal cancer. J Surg Oncol.

[24] Juloski JT, Rakic A, Cuk VV, Cuk VM, Stefanovic S, Nikolic D (2020). Colorectal cancer and trace elements alteration. J Trace Elem Med Biol.

[25] Cabral M, Kuxhaus O, Eichelmann F, Kopp JF, Alker W, Hackler J (2021). Trace element profile and incidence of type 2 diabetes, cardiovascular disease and colorectal cancer: results from the EPIC-Potsdam cohort study. Eur J Nutr.

[26] Nawi AM, Chin SF, Azhar Shah S, Jamal R (2019). Tissue and Serum Trace Elements Concentration among Colorectal Patients: A Systematic Review of Case-Control Studies. Iran J Public Health.

[27] Nawi AM, Chin SF, Mazlan L, Jamal R (2020). Delineating colorectal cancer distribution, interaction, and risk prediction by environmental risk factors and serum trace elements. Sci Rep.

[28] Pusatcioglu CK, Nemeth E, Fantuzzi G, Llor X, Freels S, Tussing-Humphreys L (2014). Systemic and tumor level iron regulation in men with colorectal cancer: a case control study. Nutr Metab (Lond).

[29] Coussens LM, Werb Z (2002). Inflammation and cancer. Nature.

[30] Hassannia B, Vandenabeele P, Vanden Berghe T (2019). Targeting Ferroptosis to Iron Out Cancer. Cancer Cell.

[31] Zheng Z, Wei Q, Wan X, Zhong X, Liu L, Zeng J (2022). Correlation Analysis Between Trace Elements and Colorectal Cancer Metabolism by Integrated Serum Proteome and Metabolome. Front Immunol.

[32] Vincenzi B, Santini D, Galluzzo S, Russo A, Fulfaro F, Silletta M (2008). Early magnesium reduction in advanced colorectal cancer patients treated with cetuximab plus irinotecan as predictive factor of efficacy and outcome. Clin Cancer Res.

[33] Wesselink E, Kok DE, Bours MJL, de Wilt JHW, van Baar H, van Zutphen M (2020). Vitamin D, magnesium, calcium, and their interaction in relation to colorectal cancer recurrence and all-cause mortality. Am J Clin Nutr.

[34] Gandevia B, Tovell A (1964). Declaration of Helsinki. Med J Aust.

[35] Zhao S, Cao S, Luo L, Zhang Z, Yuan G, Zhang Y (2018). A preliminary investigation of metal element profiles in the serum of patients with bloodstream infections using inductively-coupled plasma mass spectrometry (ICP-MS). Clin Chim Acta.

[36] Prior IA, Lewis PD, Mattos C (2012). A comprehensive survey of Ras mutations in cancer. Cancer Res.

[37] Dienstmann R, Vermeulen L, Guinney J, Kopetz S, Tejpar S, Tabernero J (2017). Consensus molecular subtypes and the evolution of precision medicine in colorectal cancer. Nature reviews Cancer.

[38] Poturnajova M, Furielova T, Balintova S, Schmidtova S, Kucerova L, Matuskova M (2021). Molecular features and gene expression signature of metastatic colorectal cancer (Review). Oncol Rep.

[39] Losi L, Ponz de Leon M, Jiricny J, Di Gregorio C, Benatti P, Percesepe A (1997). K-ras and p53 mutations in hereditary non-polyposis colorectal cancers. Int J Cancer.

[40] Huang J, Papadopoulos N, McKinley AJ, Farrington SM, Curtis LJ, Wyllie AH (1996). APC mutations in colorectal tumors with mismatch repair deficiency. Proc Natl Acad Sci U S A.

[41] Pantsar T (2020). The current understanding of KRAS protein structure and dynamics. Comput Struct Biotechnol J.

[42] Hunter JC, Manandhar A, Carrasco MA, Gurbani D, Gondi S, Westover KD (2015). Biochemical and Structural Analysis of Common Cancer-Associated KRAS Mutations. Mol Cancer Res.

[43] Lievre A, Bachet JB, Le Corre D, Boige V, Landi B, Emile JF (2006). KRAS mutation status is predictive of response to cetuximab therapy in colorectal cancer. Cancer Res.

[44] Ge XJ, Jiang JY, Wang M, Li MY, Zheng LM, Feng ZX (2020). Cetuximab enhances the efficiency of irinotecan through simultaneously inhibiting the MAPK signaling and ABCG2 in colorectal cancer cells. Pathol Res Pract.

[45] Ibrahim S, Chaigne J, Dakik H, Fourbon Y, Corset L, Lecomte T (2021). SK4 oncochannels regulate calcium entry and promote cell migration in KRAS-mutated colorectal cancer. Cell Calcium.

[46] Ge EJ, Bush AI, Casini A, Cobine PA, Cross JR, DeNicola GM (2022). Connecting copper and cancer: from transition metal signalling to metalloplasia. Nature reviews Cancer.

[47] Aubert L, Nandagopal N, Steinhart Z, Lavoie G, Nourreddine S, Berman J (2020). Copper bioavailability is a KRAS-specific vulnerability in colorectal cancer. Nat Commun.

[48] Vincenzi B, Galluzzo S, Santini D, Rocci L, Loupakis F, Correale P (2011). Early magnesium modifications as a surrogate marker of efficacy of cetuximab-based anticancer treatment in KRAS wild-type advanced colorectal cancer patients. Ann Oncol.

[49] Hu Y, McIntosh GH, Le Leu RK, Woodman R, Young GP (2008). Suppression of colorectal oncogenesis by selenium-enriched milk proteins: apoptosis and K-ras mutations. Cancer Res.

[50] Dedeurwaerdere F, Claes KB, Van Dorpe J, Rottiers I, Van der Meulen J, Breyne J (2021). Comparison of microsatellite instability detection by immunohistochemistry and molecular techniques in colorectal and endometrial cancer. Sci Rep.

[51] Gilson P, Merlin JL, Harle A (2021). Detection of Microsatellite Instability: State of the Art and Future Applications in Circulating Tumour DNA (ctDNA). Cancers (Basel).

[52] Keum N, Liu L, Hamada T, Qian ZR, Nowak JA, Cao Y (2019). Calcium intake and colon cancer risk subtypes by tumor molecular characteristics. Cancer Causes Control.

[53] Weisenberger DJ, Levine AJ, Long TI, Buchanan DD, Walters R, Clendenning M (2015). Association of the colorectal CpG island methylator phenotype with molecular features, risk factors, and family history. Cancer Epidemiol Biomarkers Prev.

[54] Slattery ML, Anderson K, Curtin K, Ma KN, Schaffer D, Samowitz W (2001). Dietary intake and microsatellite instability in colon tumors. Int J Cancer.

[55] Zhu Z, Zhao Q, Song W, Weng J, Li S, Guo T (2022). A novel cuproptosis-related molecular pattern and its tumor microenvironment characterization in colorectal cancer. Front Immunol.

[56] Kang S, Na Y, Joung SY, Lee SI, Oh SC, Min BW (2018). The significance of microsatellite instability in colorectal cancer after controlling for clinicopathological factors. Medicine (Baltimore).

