# Supplementary material for: The Contributions of Trace Elements on Molecular Subtype-Specific Colorectal Cancer
Source: J Cancer. 2023 May 21;14(9):1486–98. doi: 10.7150/jca.81686 (PMC10266256; doi:10.7150/jca.81686)
Supplement: Supplementary file 1 — Supplementary figures. [file jcav14p1486s1.pdf]

The Contributions of Trace Elements on Molecular Subtype-Specific Colorectal Cancer

Dong-Xiao Bai, MD[1]; Jian-an Xiao, BS[1]; Tian-Chen Huang, PhD[1]; Zhi-Ling Shen, BS[1]; Lei Li, MD[1]; Fei-Fei Ding, MD[2]; Ming Wen, MD[2]; Shou-Xin Wu, PhD[2]; Xiao-Chen Liu, BS[3*]✉, Hui-Hui Jiang, PhD[2*]✉

1. The Fourth Department of General Surgery, Anyang Tumor Hospital, The Affiliated Anyang Tumor Hospital of Henan University of Science and Technology, Anyang 455000, China.
2. Zhangjiang Center for Translational Medicine, Shanghai Biotecan Pharmaceuticals Co., Ltd., Shanghai 200021, China.
3. Department of General Surgery, Affiliated Three Two Zero one Hospital of Xi`an Jiaotong University, Hanzhong 723099, China.

✉ Corresponding authors: Huihui Jiang (phone: 86-021-50277725; e-mail: jianghh@biotecan.com) and Xiaochen Liu (phone: 86-0916-2383462; e-mail: 13509165562@163.com).

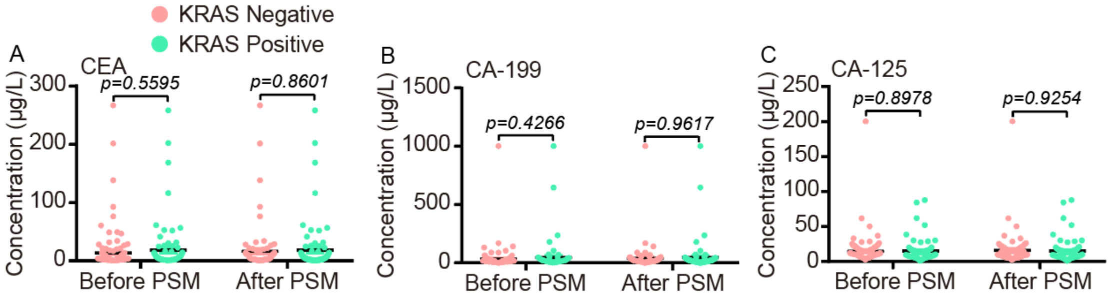

Figure S1. The comparative analysis of 3 traditional biomarkers between the K-RAS positive group and the K-RAS negative group before or after PSM. Statistical analysis was performed by the Two-tailed Mann-Whitney U test.

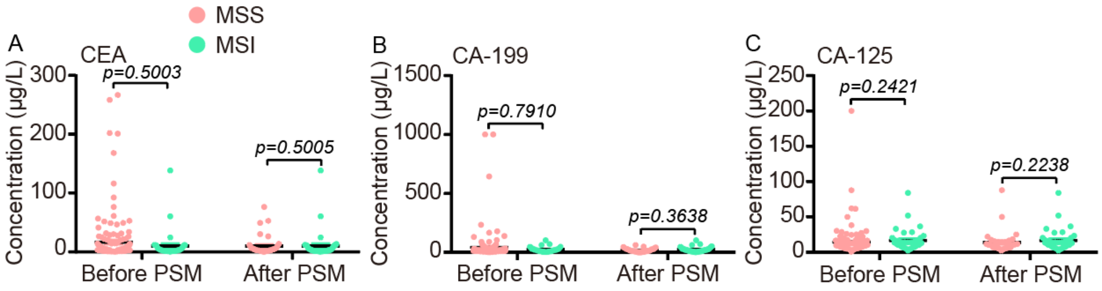

Figure S2. The comparative analysis of 3 traditional biomarkers between the MSS group and the MSI group before or after PSM. Statistical analysis was carried out by the Two-tailed Mann-Whitney U test.
